# miR-29c plays a suppressive role in breast cancer by targeting the TIMP3/STAT1/FOXO1 pathway

**DOI:** 10.1186/s13148-018-0495-y

**Published:** 2018-05-16

**Authors:** Wan Li, Jie Yi, Xiangjin Zheng, Shiwei Liu, Weiqi Fu, Liwen Ren, Li Li, Dave S. B. Hoon, Jinhua Wang, Guanhua Du

**Affiliations:** 1 The State Key Laboratory of Bioactive Substance and Function of Natural Medicines, Beijing, China; 2 Key Laboratory of Drug Target Research and Drug Screen, Institute of Materia Medica, Chinese Academy of Medical Science and Peking Union Medical College, Beijing, 100050 China; 3 Department of Clinical Laboratory, Peking Union Medical College Hospital, Beijing, 100730 China; 4 Department of Endocrinology, Shanxi DAYI Hospital, Shanxi Medical University, Taiyuan, 030002 Shanxi China; 5 Department of Translational Molecular Medicine, John Wayne Cancer Institute (JWCI) at Providence Saint John’s Health Center, Santa Monica, CA 90404 USA

**Keywords:** Breast cancer, miR-29c, DNMT3B, methylation, and STAT1/FOXO1

## Abstract

**Background:**

miR-29c has been associated with the progression of many cancers. However, the function and mechanism of miR-29c have not been well investigated in breast cancers.

**Methods:**

Real-time quantitative PCR was used to assess expression of miR-29c and DNMT3B mRNA. Western blot and immunochemistry were used to examine the expression of DNA methyltransferase 3B (DNMT3B) protein in breast cancer cells and tissues. The functional roles of miR-29c in breast cancer cells such as proliferation, migration, invasion, colony formation, and 3D growth were evaluated using MTT, transwell chambers, soft agar, and 3D Matrigel culture, respectively. In addition, the luciferase reporter assay was used to check if miR-29c binds the 3′UTR of DNMT3B. The effects of miR-29c on the DNMT3B/TIMP3/STAT1/FOXO1 pathway were also examined using Western blot and methyl-specific qPCR. The specific inhibitor of STAT1, fludarabine, was used to further check the mechanism of miR-29c function in breast cancer cells. Studies on cell functions were carried out in DNMT3B siRNA cell lines.

**Results:**

The expression of miR-29c was decreased with the progression of breast cancers and was closely associated with an overall survival rate of patients. Overexpression of miR-29c inhibited the proliferation, migration, invasion, colony formation, and growth in 3D Matrigel while knockdown of miR-29c promoted these processes in breast cancer cells. In addition, miR-29c was found to bind 3′UTR of DNMT3B and inhibits the expression of DNMT3B, which was elevated in breast cancers. Moreover, the protein level of TIMP3 was reduced whereas methylation of TIMP3 was increased in miR-29c knockdown cells compared to control. On the contrary, the protein level of TIMP3 was increased whereas methylation of TIMP3 was reduced in miR-29c-overexpressing cells compared to control. Knockdown of DNMT3B reduced the proliferation, migration, and invasion of breast cancer cell lines. Finally, our results showed that miR-29c exerted its function in breast cancers by regulating the TIMP3/STAT1/FOXO1 pathway.

**Conclusion:**

The results suggest that miR-29c plays a significant role in suppressing the progression of breast cancers and that miR-29c may be used as a biomarker of breast cancers.

**Electronic supplementary material:**

The online version of this article (10.1186/s13148-018-0495-y) contains supplementary material, which is available to authorized users.

## Background

Cancer is a major public health problem worldwide and is the second leading cause of death in China and the USA [1, 2]. Breast cancer is the most commonly diagnosed female cancer in the world. It is extremely important to elucidate the mechanisms of breast cancer development and progression and to facilitate early diagnosis.

In the last decades, studies have focused on the epigenetic changes in the development and progression of breast cancers. Epigenetic changes include DNA methylation, histone modifications, abnormal expression of non-coding RNAs, and chromatin remodeling [3]. microRNAs are conserved small non-coding RNAs that regulate gene expression at the translational or post-transcriptional level by repressing or degrading target messenger RNAs [4]. miRNAs are widely expressed in normal tissues and are often miss-regulated in disease states [5]. There is increasing evidence that miRNAs play critical roles in tumorigenesis by functioning as oncogenes or tumor suppressors [6]. Recently, miRNAs have emerged as novel biomarkers in the diagnosis, therapy, and prevention of breast cancer. The miR-29 family consists of miR-29a, miR-29b, and miR-29c. They regulate a series of biological processes, including cell proliferation [7], epigenetic modification [8], intracellular signaling [9], and cell movement [10]. The expression of miR-29c is decreased in many cancers including hepatocellular carcinoma [11], leukemia [12], glioma [13], bladder cancer [14], gastric carcinoma [15], breast cancer [16, 17], and melanoma [18].

DNA methylation is an epigenetic modification that is involved in many of the vital biological functions, such as embryonic development [19], regulation of gene expression [20], imprinting [21], and X-chromosome inactivation [22]. DNMT3B, one of the major DNA methyltransferases, is thought to function in de novo methylation of DNA [23]. DNA methylation of promoter CpG dinucleotides is associated with the suppression of gene expression in cancers. Several studies have shown that DNMT3B is frequently upregulated and plays an important role in many cancers [24, 25]. It is unclear if there is an association between miR-29c and DNMT3B, and what regulatory mechanisms of miR-29c and DNMT3B occur in breast cancer cells.

In this study, we investigated the expression of miR-29c in breast cancer at different clinicopathological stages to identify any association with clinical staging and overall patient survival. We also studied the effects of miR-29c overexpression in cell growth, migration, invasion, and colony formation. The binding of miR-29c to the 3′UTR of DNMT3B was examined, and the effects of knockdown of DNMT3B on proliferation, migration, and invasion were investigated in breast cancer cell lines. The expression of DNMT3B, TIMP3, STAT1, and FOXO1 was assessed by Western blot in miR-29c overexpression cells, miR-29c knockdown cells, and DNMT3B siRNA cells. Together, the results demonstrate a suppressive role of miR-29c by targeting the TIMP3/STAT1/FOXO1 pathway in breast cancer cell proliferation, migration and invasion, and suggest that the loss of miR-29c might be a novel biomarker related to the progression of breast cancer.

## Methods

### Cell culture

MCF-7, MDA-MB-231, and MDA-MB-436 breast cancer cell lines were purchased from the Cell Bank of the Chinese Academy of Sciences (Beijing, China). All cells lines were authenticated using the Short Tandem Repeat (STR) method, performed by this cell bank. MCF-7 cells were maintained in RPMI 1640 (Thermo Fisher Scientific, Carlsbad, CA) while MDA-MB-231 and MDA-MB-436 cells were cultured in Dulbecco’s modified Eagle medium (DMEM, Thermo Fisher Scientific). The complete culture medium was prepared by adding 10% fetal bovine serum (FBS, Thermo Fischer Scientific). Cells were cultured at 37 °C in a humidified incubator containing 5% CO_2_.

For drug treatment, cells were treated with fludarabine 2 μM, a STAT1 inhibitor for 24 h and used in specific cell culture experiments.

### Breast cancer tissues

The use of human tissues was approved by the Institutional Review Board at the Peking Union Medical College Hospital, Beijing, China. Breast cancer tissues were diagnosed at a different stage and obtained from the Peking Union Medical College Hospital Pathology Department.

### miRNA was extracted from serums

Serum samples were collected from 20 healthy females and 79 breast cancer patients at a different stage. The use of serums was also approved by the Institutional Review Board at the Peking Union Medical College Hospital, Beijing, China. Cell-free total RNA including primary miRNA and other small RNA was purified from serum by using miRNeasy Serum/Plasma Kit (Cat No 217184, QIAGEN, Hilden, China).

### Transfection

MCF-7, MDA-MB-231, and MDA-MB-436 cells were plated in 60-mm dishes at 80% confluence before transfection. Anti-miR-29c (Cat# AM17000, Thermo Fisher Scientific, Waltham, MA USA) was transfected into MCF-7 cells, and miR-29c mimic (Cat# 4464066, Thermo Fisher Scientific, Waltham, MA, USA) was transfected into MDA-MB-231 and MDA-MB-436 cells using the Lipofectamine™ 3000 transfection reagent (Invitrogen, Grand Island, NY). After transfection for 48 h, the cells were collected and used for the in vitro functional analysis.

MDA-MB-231 miR-29c cells (after transfection of miR-29c mimic for 48 h) were transfected with DNMT3B expression plasmid, and the functions of cells with high DNMT3B were evaluated in following experiments including migration, invasion, colony formation, and 3D Matrigel growth assays.

DNMT3B siRNA 1 and DNMT3B siRNA 2 (Integrated DNA Technologies, Inc., Coralville, IA) were also transfected into MDA-MB-231 and MDA-MB-436 cells. Sequences of DNMT3B siRNA 1 and 2 can be seen in Additional file 1: Table S1. After transfection for 48 h, cells were collected and used for the in vitro cell culture experiments listed below.

### Cell proliferation assay

Cells which were transfected with anti-miR-29c and miR-29c mimic (Origene, Rockville, MD) were seeded in 96-well plates at 2 × 10^3^/well, respectively, and cultured for 24, 48, 72, and 96 h. Ten microliters CCK-8 (Dojindo, Kumamoto, Japan) was added to the cells for 3–4 h, and their viability was measured at 450 nm using SpectraMax M5 Microplate Reader, according to the manufacturer’s instructions.

### Cell migration and invasion assays

Briefly, 10^4^ cells were plated on the top of transwells with 8.0-μm pore polycarbonate membrane inserts (Corning, New York, NY) for the migration assay. For the invasion assay, the inserts were coated with a thin layer of Matrigel basement membrane matrix (BD Biosciences San Diego, CA). Serum (10%) was used as the chemoattractant. After 24 h, the cells on the lower surface of the inserts were fixed with methanol for 15 min, stained with 1% crystal violet solution for 15 min, and counted using a light microscope.

### Soft agar colony formation assay and 3D Matrigel culture

The soft agar colony formation assay was performed using 6-well plates. Each well contained 2 mL of 0.7% agar in complete medium as the bottom layer and 1 mL of 0.35% agar in complete medium containing 3000 cells as the top layer. Cultures were then maintained under standard culture conditions for 3–4 weeks. After culture, the colonies were stained with MTT solution (200 μL/well), and the number of clones was counted. The 3D cell culture was performed using Matrigel matrix (BD Biosciences, San Diego, CA) in 96-well plates. Each well contained a mixture of 50 μL complete medium containing 1000 cells and 50 μL Matrigel matrix.

### In silicon assay

To explore the expression of DNMT3B in breast cancer, in silicon assay was carried out using data from Oncomine (www.oncomine.org) and TCGA. The database from Oncomine is very useful for investigating genes that are expressed in multiple cancer datasets to validate the relationship between transcription and disease. More advanced analyses were used to check the expression of genes in a small fraction of samples of a cancer type using different filters.

To explore if survival rate of patients with breast cancers was associated with miR-29c or DNMT3B. Data online (OncoLnc: linking TCGA survival data to mRNAs, miRNAs, and lncRNAs) was used. OncoLnc is available at http://www.oncolnc.org. The plot of Kaplan-Meier was automatically given using TCGA data when the special gene was decided and values of lower and higher percentiles were input [26].

### Western blot

Whole cell extracts were prepared from MCF-7 control and MCF-7 transfected with anti-miR-29c, MDA-MB-231 control and MDA-MB-231 transfected with miR-29c mimic, and MDA-MB-436 control and MDA-MB-436 transfected with miR-29c mimic and MDA-MB-231 transfected with DNMT3B siRNAs. In brief, cells were lysed in radioimmunoprecipitation assay (RIPA) lysis buffer at 4 °C for 30 min. The cell lysate was centrifuged at 12,000 rpm for 10 min at 4 °C, and supernatant was collected. Protein concentrations were determined by using the BCA Kit (Beyotime, Guangzhou, China). Cell extracts were resolved by 10% SDS-PAGE, and the separated proteins were transferred to a polyvinylidene difluoride membrane (Millipore, Billerica, MA). The membranes were blocked in 5% fat-free milk in TBS containing 0.1% Tween 20 for 1 h and then immunoblotted with primary antibodies with appropriate dilutions overnight at 4 °C. The primary antibodies for staining included DNMT3B (Abcam, Cambridge, MA), TIMP3 (EMD Millipore, Billerica, MA), STAT1 (Cell Signal, Boston, MA), and FOXO1 (Cell Signal, Boston, MA). After immunoblotting, the membranes were washed three times with phosphate-buffered saline with Tween (PBST) and followed by 1-h incubation with horseradish peroxidase-conjugated goat anti-rabbit Ab (1:5000, Santa Cruz Biotech, Santa Cruz, CA) or horseradish peroxidase-conjugated rabbit anti-mouse Ab (1:5000, Santa Cruz). The densities of protein bands were quantified by Alpha Ease FCTM software (Version 3.1.2, Alpha Innotech Corp, San Leandro, CA).

### Quantitative reverse transcription PCR

Total extracted RNA (1 μg) was used for cDNA synthesis, with Oligo(dT) 20 primers (Invitrogen, Grand Island, NY). The cDNA was added to a quantitative reverse transcription-PCR mixture that contained 1× SYBR Green PCR master mix (Quanta Biosciences, Gaithersburg, MD) and 500 nmol/L gene-specific primers. Assays were performed in triplicate on a CFX thermocycler (Bio-Rad, Hercules, CA). The primers are listed in Additional file 1: Table S2.

### Quantitative real-time methylation-specific PCR

The extraction of genomic DNA from breast cancer cells (1 × 10^6^) was performed using a QIAamp DNA Mini Kit (Qiagen, Hilden, Germany) according to the manufacturer’s protocol. Bisulfite modification was strictly carried out according to the manufacturer’s instructions (Qiagen). The methylation status of TIMP3 in specimens was assessed by quantitative real-time methylation-specific PCR (qMSP) using two sets of primers designed for methylated (M) or unmethylated (U) DNA sequences using bisulfite-modified DNA. The methylation-specific primers and unmethylated-specific primers for the TIMP3 gene were designed according to the standard methods as described previously [27] and are listed in Additional file 1: Table S3.

### Immunohistochemistry

Five-micrometer paraffin-embedded tissue sections (35 tissues of breast cancers and 20 adjacent normal tissues) were deparaffinized and rehydrated, antigens were retrieved, and IHC procedures were performed as reported previously [28]. Briefly, the deparaffinized sections were immersed in boiled 10 mM sodium citrate buffer (pH 6.0) and maintained at sub-boiling temperature for 20 min. After that, peroxidase activity was inactivated by incubation with 3% H_2_O_2_ solution for 10 min at room temperature. Then, the sections were incubated with primary DNMT3B antibody (1:50 dilution; Abcam, Cambridge, MA) in a moist chamber at 4 °C overnight. Negative controls were conducted by the exchange of primary antibody for PBS. Slides were incubated with biotinylated anti-rabbit immunoglobulins for 60 min at room temperature and treated with streptavidin-peroxidase (DAKO, Woodbridge, VA). Staining was achieved by 3,3-diaminobenzidine (DAB; Vectastain, Vector Laboratories, Inc., Linaris GmbH), and the slides were counterstained with hematoxylin. Photographs were taken with equal exposure on a Nikon Eclipse Ti microscope coupled with NIS elements software (Nikon, Melville, NY) for Windows. Three visual fields were randomly selected for the quantitative analysis which was performed independently by two pathologists. The semi-quantitative analysis of the stained sections was done by light microscopy according to the immunoreactive score of Remmele and Stegner (IRS) [29, 30].

### Luciferase reporter assay

DNMT3B luciferase reporters containing DNMT3B wild-type 3′UTR (CUCUUCUUACUGGUGCUA) or mutant 3′UTR (CUCUUCUUACUCCUCCUA) were purchased from SWITCH Gear Genomics Company (Menlo Park, CA). MDA-MB-231 cells were co-transfected with the 50 ng luciferase reporter, 1 ng Renilla luciferase reporter (pRL-CMV vector, Promega, Madison, WI), or/and 100 nM mimic miR-29c by Lipofectamine 3000 (Invitrogen, Grand Island, NY), respectively. After transfection for 24 h, cells were lysed and centrifuged at 12000 rpm for 10 min. The supernatant was collected following centrifuging, and luciferase activities were measured using the Dual-Luciferase Assay System (Promega, Madison, WI). The activity of Renilla luciferase was normalized to that of firefly luciferase.

### Statistical analysis

The results are given as mean ± SD. Student’s *t* test was used to calculate the differences between the two study groups. One-way ANOVA followed by LSD test was used to calculate the differences among multiple study groups. Fisher’s exact test was used to calculate the proportional differences of immunoreactive scores between normal and tumor samples. Differences were considered statistically significant at *P* < 0.05.

## Results

### The expression level of miR-29c was reduced in breast cancer and was positively correlated with patient survival rate

**Fig. 1:**
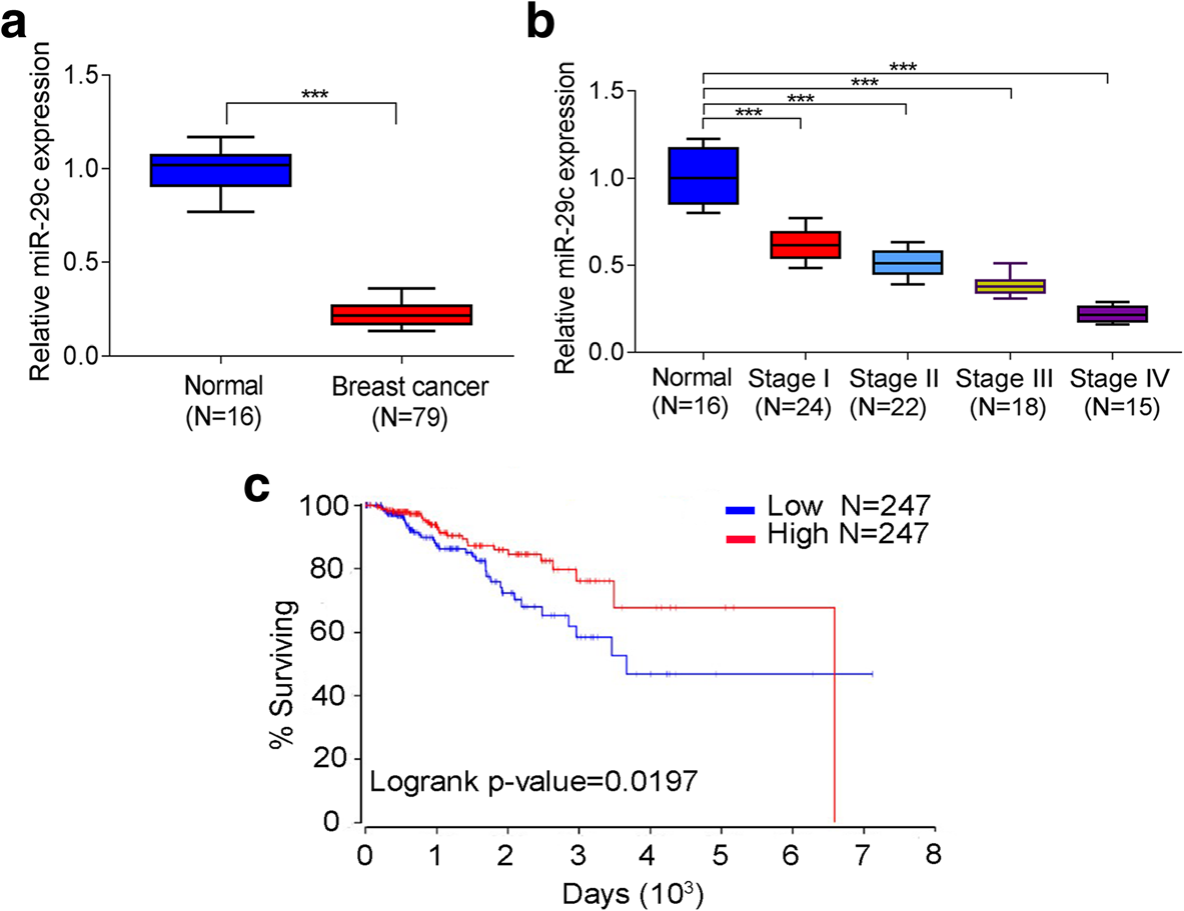
The expression of miR-29c was reduced in breast cancers and was positively correlated with the survival rate of breast cancer patients. **a** The expression of miR-29c in normal tissues and breast cancer tissues was checked by qRT-PCR. **b** The expressions of miR-29c in the serum of normal controls and breast cancer patients at different stages were evaluated by qRT-PCR. **c** Kaplan-Meier analysis of overall survival curves for breast cancer patients with low versus high expressions of miR-29. Data were presented as mean ± SD, ***P < 0.001

To assess the expression of miR-29c in breast cancer and normal tissues, we extracted mRNA from breast cancer tissues and normal tissues and checked the expression of miR-29c by qRT-PCR. As shown in Fig. 1a, the expression of miR-29c was much lower in breast cancers than in normal tissues. We also examined the expression of miR-29c in serum from breast cancer patients at different stages and found that the expression of miR-29c in the serum was decreased with the progression of breast cancers (Fig. 1b). Furthermore, Kaplan-Meier meta-analyses of miR-29c using online TCGA data (http://www.oncolnc.org) showed that patients with high miR-29c expression had a higher survival rate than patients with low miR-29c expression, respectively (Fig. 1c)*.*

### Level of DNMT3B expression was upregulated in breast cancer tissues and negatively correlated with the survival rate

**Fig. 2:**
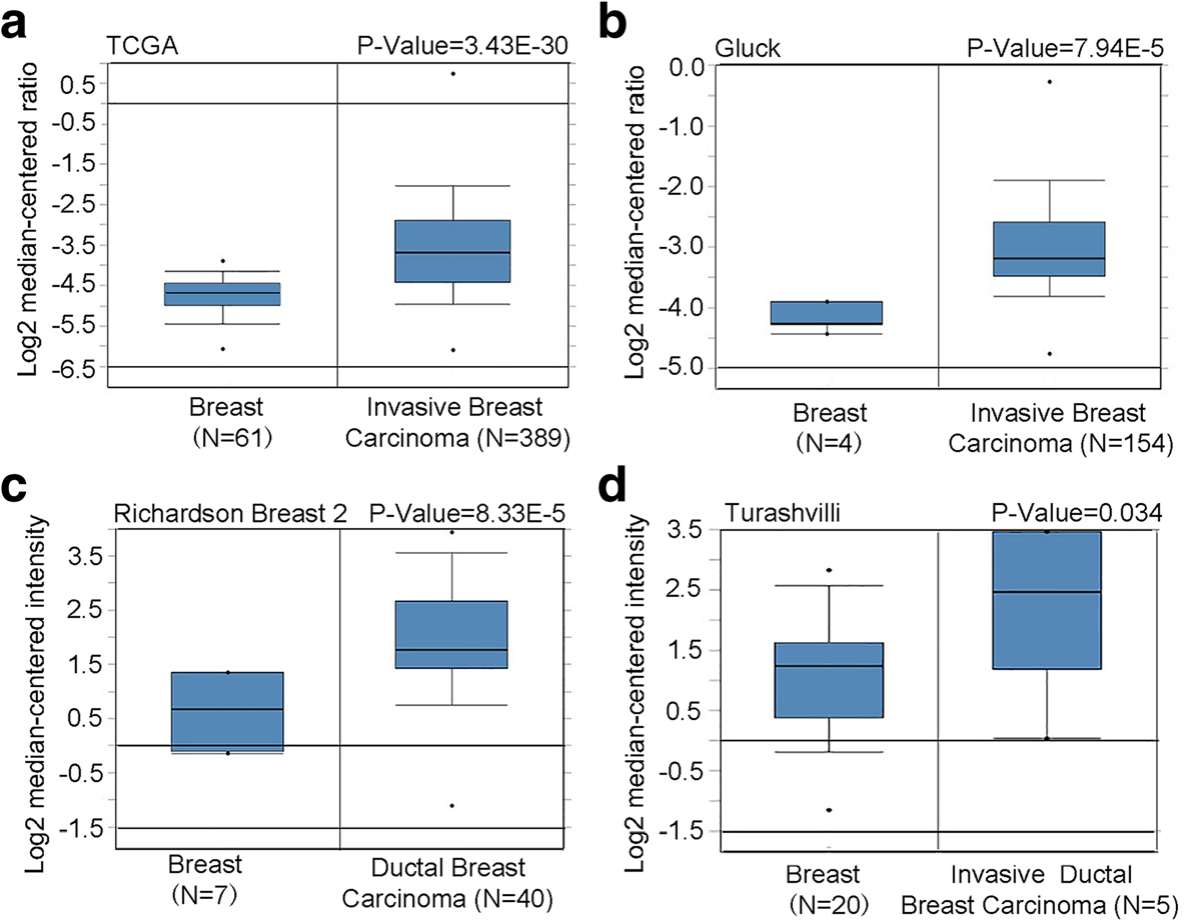
The data retrieved from Oncomine and TCGA showed that the expression of DNMT3B mRNA was increased in breast cancers. **a** Comparison of the mRNA levels of DNMT3B in normal breast tissues (*N* = 61) and invasive breast carcinoma tissues (*N* = 389) using TCGA data retrieved from the Oncomine database. **b** Comparison of the mRNA levels of DNMT3B in normal breast tissues (*N* = 4) and invasive breast carcinoma tissues (*N* = 154) using Gluck’s data retrieved from the Oncomine database. **c** Comparison of the mRNA levels of DNMT3B in normal breast tissues (*N* = 7) and ductal breast carcinoma tissues (*N* = 40) using Richardson’s data retrieved from the Oncomine database. **d** Comparison of the mRNA levels of DNMT3B in normal breast tissues (*N* = 20) and invasive ductal breast carcinoma tissues (*N* = 5) using Turashvili’s data retrieved from the Oncomine database

To investigate the expression of DNMT3B mRNA in breast cancer tissues, publicly available expression data for DNMT3B were retrieved from Oncomine and TCGA. Results showed that the expression level of DNMT3B mRNA was upregulated in invasive breast carcinoma (Fig. 2a, b), ductal breast carcinoma (Fig. 2c), and invasive ductal breast carcinoma (Fig. 2d).

**Fig. 3:**
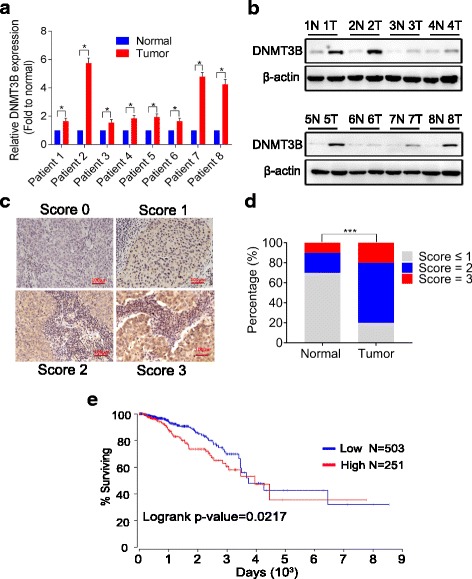
DNMT3B expression was upregulated in breast cancers and was negatively correlated with the survival rate of breast cancer patients. **a** mRNA levels of DNMT3B detected by qRT-PCR in eight breast cancer tissues were higher than that in their adjacent normal tissues. **b** Comparison of the protein levels of DNMT3B in eight paired breast cancer and adjacent non-tumor tissues by Western blotting. **c** Immunohistochemistry representative photos of DNTM3B expression in normal and different immunoreactive scores breast cancer tissues. **d** Immunoreactive scores of DNMT3B in breast cancer and the adjacent normal tissues. **e** Kaplan-Meier analysis of overall survival curves for breast cancer patients with low versus high expression of DNMT3B. Data were presented as mean ± SD, *P < 0.05, ***P < 0.001

The expression of DNMT3B mRNA and protein in eight breast cancer tissues and eight adjacent non-tumor tissues was assessed by qRT-PCR and Western blot. As shown in Fig. 3a, b and Additional file 1: Figure S1, the expression level of DNMT3B mRNA and protein, respectively, was higher in breast cancers than in normal breast tissues. To check the protein expression in breast cancer tissues, IHC was carried out, and the results showed that DNMT3B expression was higher in breast cancers than that in normal breast tissues (Fig. 3c, d). In addition, we performed in silico analysis using an online TCGA database (http://www.oncolnc.org) and found that breast cancer patients with high DNMT3B mRNA expression had a lower survival rate than patients with low DNMT3B mRNA expression (Fig. 3e).

### miR-29c inhibited the proliferation, migration, and invasion of breast cancer cells, which could be reversed by the overexpression of DNMT3B

**Fig. 4:**
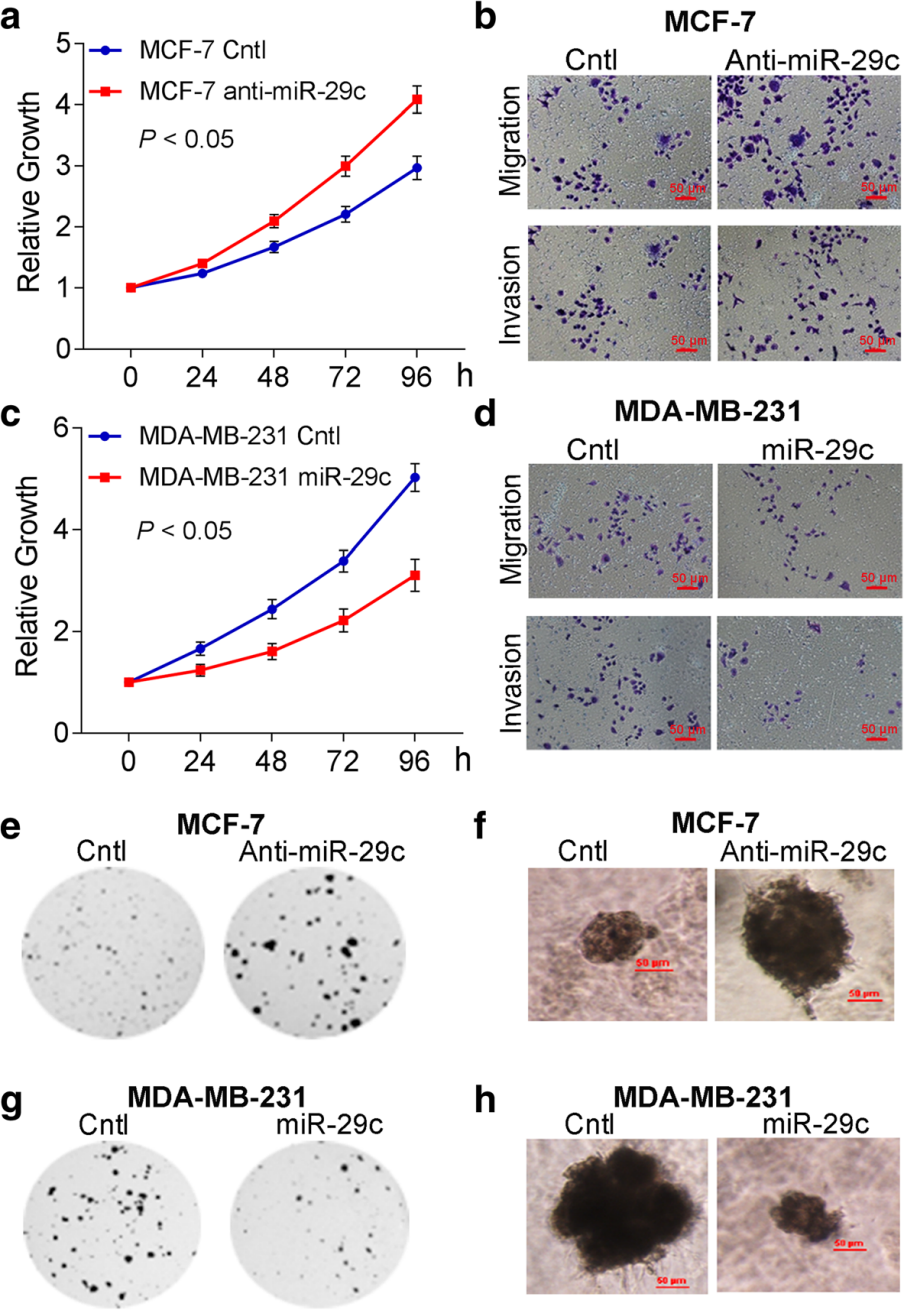
miR-29c inhibited the proliferation, migration and invasion, colony formation, and growth in 3D Matrigel of breast cancer cells. **a** Proliferation of MCF-7 anti-miR-29c is higher than that of MCF-7 Cntl by CCK8 proliferation assay. **b** Migration and invasion of MCF-7 anti-miR-29cis higher than that of MCF-7 Cntl. **c** Proliferation of MDA-MB-231 miR-29c mimic is lower than that MDA-MB-231 Cntl by CCK8 proliferation assay. **d** Migration and invasion assays of MDA-MB-231 miR-29c mimic are lower than that MDA-MB-231 Cntl. **e** Colony formations of MCF-7 anti-miR-29c are more than that of MCF-7 Cntl in Soft agar assays. **f** Growth of MCF-7 anti-miR-29c is more than that of MCF-7 Cntl in 3D Matrigel culture. **g** Colony formations of MDA-MB-231 miR-29c mimic are less than that of MDA-MB-231 Cntl in soft agar assays. **h** Growth of MDA-MB-231 miR-29c mimic is less than that of MDA-MB-231 Cntl in 3D Matrigel culture. Data are presented as mean ± SD from three independent experiments, and every experiment was repeated three times, *P < 0.05

To explore the functional role of miR-29c in breast cell lines, we performed loss- and gain-of-function analysis in breast cancer cells. The knockdown of miR-29c in MCF-7 cells increased cell proliferation (Fig. 4a) and promoted the migration and invasion of cells (Fig. 4b, Additional file 1: Figure S2). In contrast, the overexpression of miR-29c in MDA-MB-231 and MDA-MB-436 cells reduced cell proliferation (Fig. 4c and Additional file 1: Figure S3) and inhibited the migration and invasion of cells (Fig. 4d, Additional file 1: Figures S2 and S3).

To determine whether miR-29c-induced inhibition of cell migration and invasion could be reversed by restoration of DNMT3B expression, we performed gain-of-function analysis in MDA-MB-231 miR-29c cells. Results showed that the overexpression of DNMT3B in MDA-MB-231 miR-29c cells promoted cell migration and invasion of cells (Additional file 1: Figure S4), which suggested that miR-29c-induced inhibition of cell migration and invasion could be reversed by the overexpression of DNMT3B.

### miR-29c inhibited colony formation and growth in breast cancer cells

To investigate the role of miR-29c in the tumorigenesis of breast cancer cells, soft agar and 3D Matrigel culture of MCF-7 Cntl, MCF-7 anti-miR-29c, MDA-MB-231 Cntl, MDA-MB-231 miR-29c, MDA-MB-436Cntl, and MDA-MB-436 miR-29c were carried out. Results showed that the knockdown of miR-29c in MCF-7 cells facilitated the colony formation and growth (Fig. 4e, f and Additional file 1: Figure S5), whereas the overexpression of miR-29c in MDA-MB-231 and MDA-MB-436 cells reduced the colony formation and growth in both soft agar (Fig. 4g, Additional file 1: Figures S3 and S5) and 3D Matrigel cultures (Fig. 4h and Additional file 1: Figure S3).

To determine whether miR-29c-induced the inhibition of tumorigenesis could be reversed by restoration of DNMT3B expression, we performed gain-of-function analysis in MDA-MB-231 miR-29c cells. The overexpression of DNMT3B in MDA-MB-231 miR-29c cells facilitated the colony formation and growth in both soft agar and 3D Matrigel cultures (Additional file 1: Figure S4), which suggested that miR-29c-induced inhibition of colony formation and growth in 3D Matrigel could be reversed by the overexpression of DNMT3B.

### DNMT3B is directly targeted by miR-29c

**Fig. 5:**
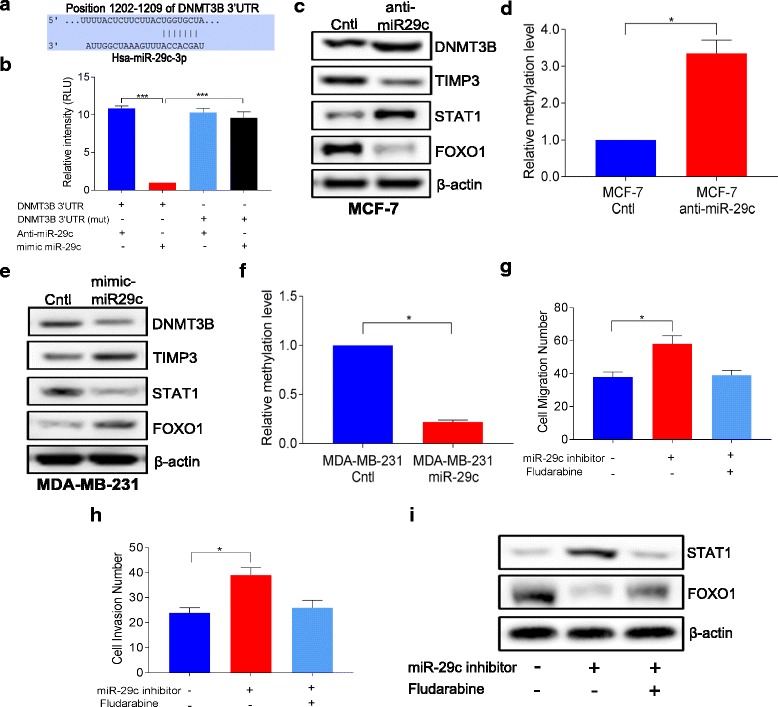
miR-29c directly targeted DNMT3B and regulated the DNMT3B/TIMP3/STAT1/FOXO1 pathway in breast cancer cells. **a** The potential binding site of miR-29c in the 3′UTR of DNMT3B. **b** Dual-Luciferase Reporter Assay of miR-29c and DNMT3B in MDA-MB-231 cells. **c** Protein levels of DNMT3B, TIMP3, STAT1, and FOXO1 detected by Western blotting in MCF-7 cells after the transfection of miR-29c inhibitor. **d** qMS-PCR assay of the methylation level of TIMP3 in MCF-7 cells after the transfection of miR-29c inhibitor. **e** Protein levels of DNMT3B, TIMP3, STAT1, and FOXO1 detected by Western blotting in MDA-MB-231 cells after the transfection of miR-29c mimic. **f** qMS-PCR assay of the methylation level of TIMP3 in MDA-MB-231 cells after the transfection of miR-29c mimic. **g** Migration assays of MCF-7 cells that were co-treated with fludarabine (STAT1 inhibitor) and miR-29c inhibitor. **h** Invasion assays of MCF-7 cells that were co-treated with fludarabine (STAT1 inhibitor) and miR-29c inhibitor. **i** Protein levels of STAT1 and FOXO1 detected by Western blotting in MCF-7 cells that were co-treated with fludarabine (STAT1 inhibitor) and miR-29c inhibitor. Data are presented as mean ± SD from three independent experiments, and every experiment was repeated three times, *P < 0.05, ***P < 0.001

Previous study showed that miR-29c was negatively correlated with the expression of DNMT3B in melanoma in our group [18]. In breast cancers, DNMT3B was also post-transcriptionally regulated by miRNAs [17, 31]. To explore the relationship between DNMT3B and miR-29c, an in silico assay was performed to investigate whether miR-29c could bind to the 3′UTR of DNMT3B (http://www.targetscan.org/, http://www.mirbase.org). This analysis identified a conserved sequence UGGUGCU is the 3′UTR as a potential binding site for miR-29c (Fig. 5a). To further verify that DNMT3B is the target of miR-29c, luciferase reporter plasmids containing conserved or mutated sequences UCCUCCU of DNMT3B were co-transfected with miR-29c mimic in MDA-MB-231 cells. As shown in Fig. 5b, the luciferase activity caused by DNMT3B 3′UTR (wild-type) was significantly reduced by miR-29c, while the luciferase activity caused by DNMT3B 3′UTR (mutation) was not reduced by miR-29c. The luciferase assay was also carried out in MCF-7 cell, and similar results were found (shown in Additional file 1: Figure S6). The expression of DNMT3B protein was reduced by overexpression of miR-29c while the expression of DNMT3B protein was increased by inhibition of miR-29c expression (Fig. 5c, e). These results suggest that miR-29c binds the 3′UTR of DNMT3B and regulates the expression of DNMT3B.

### miR-29c inhibits breast cancer cells by targeting the DNMT3B/TIMP3/STAT1/FOXO1 pathway

TIMP3 plays a role as a tumor suppressor in many cancers. Methylation of TIMP3 has been found in several cancers [32, 33]. Since DNMT3B is a major DNA methyltransferase, regulated by miR-29c, we investigated TIMP3 methylation and the expression in MDA-MB-231 cells with miR-29c overexpression and MCF-7 cells with miR-29c knockdown. Methylation of TIMP3 was increased, whereas the protein level of TIMP3 was reduced in MCF-7 cells with miR-29c knockdown compared to control (Fig. 5c, d, Additional file 1: Figure S7B). On the contrary, methylation of TIMP3 was reduced, and the protein level of TIMP3 was increased in MDA-MB-231 with miR-29c overexpression compared to control (Fig. 5e, f, Additional file 1: Figure S7F). STAT1 and FOXO1 are downstream targets of TIMP3. To confirm that miR-29c exerts its function on breast cancer by STAT1/FOXO1 pathway, the protein levels of STAT1 and FOXO1 were checked in MDA-MB-231 cells with miR-29c overexpression and MCF-7 cells with miR-29c knockdown. As shown in Fig. 5c, e and Additional file 1: Figures S7C and S5D, the protein expression of STAT1 was increased while the expression of FOXO1 was reduced in MCF-7 cells with miR-29c knockdown compared to control. On the contrary, the protein expression of STAT1 was reduced while the protein expression of FOXO1 was increased in MDA-MB-231 with miR-29c overexpression compared to control (Fig. 5c, e, Additional file 1: Figures S7G and S7H. In addition, pretreatment of cells with fludarabine, a specific inhibitor of STAT1 activation but not of other STATs, abrogated the increment of migration and invasion caused by miR-29c inhibition in MCF-7 cells (Fig. 5g, h). Furthermore, pretreatment of fludarabine blocked the changes in expression of STAT1 and FOXO1 caused by miR-29c inhibition in MCF-7 cells (Fig. 5i). All together, these results suggest that miR-29c inhibits breast cancer by targeting the DNMT3B/TIMP3/STAT1/FOXO1 pathway (Fig. 7).

### Knockdown of DNMT3B by siRNA reduced the proliferation, migration, and invasion

**Fig. 6:**
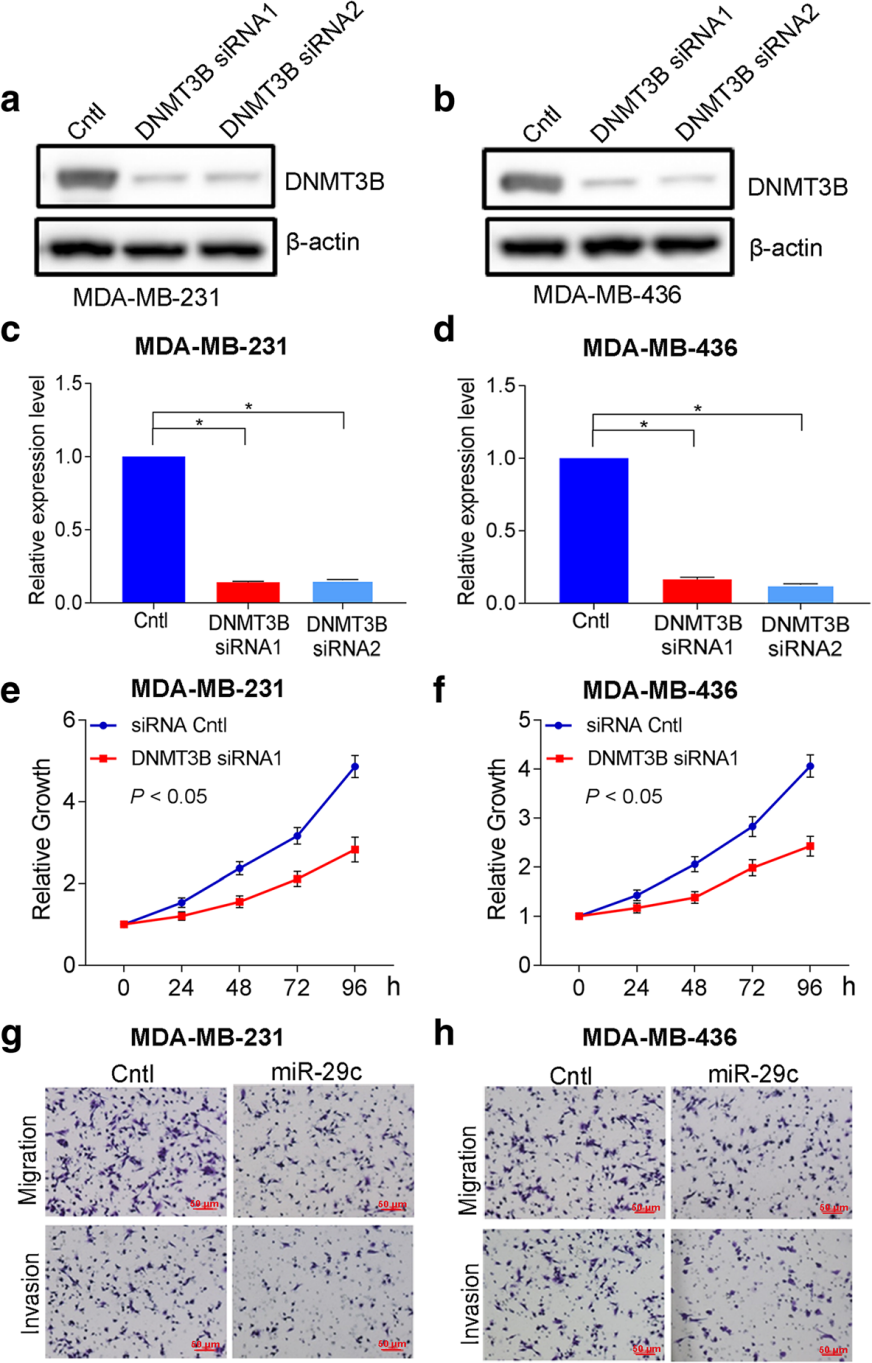
Knockdown of DNMT3B inhibited the proliferation, migration, and invasion in MDA-MB-231 and MDA-MB-436 cells. **a** Protein levels of DNMT3B detected by Western blotting in MDA-MB-231 cells after the transfection of DNMT3B siRNA 1 and 2. **b** Protein levels of DNMT3B detected by Western blotting in MDA-MB-436 cells after the transfection of DNMT3B siRNA 1 and 2. **c** Quantification of DNMT3B protein levels in MDA-MB-231 cells after the transfection of DNMT3B siRNA 1 and 2. **d** Quantification of DNMT3B protein levels in MDA-MB-436 cells after the transfection of DNMT3B siRNA 1 and 2. **e** CCK-8 proliferation assays of MDA-MB-231 cells after the transfection of DNMT3B siRNA. **f** CCK-8 proliferation assays of MDA-MB-436 cells after the transfection of DNMT3B siRNA. **g** Migration and invasion assays of MDA-MB-231 cells after the transfection of DNMT3B siRNA. **h** Migration and invasion assays of MDA-MB-436 cells after the transfection of DNMT3B siRNA. Data are presented as mean ± SD from three independent experiments, and every experiment was repeated three times

To check whether DNMT3B protein expression affected cell proliferation, migration, and invasion, MDA-MB-231 and MDA-MB-436 were transfected with DNMT3B siRNAs. As shown in Fig. 6a–d, DNMT3B protein expression was significantly reduced by both DNMT3B siRNA 1 and DNMT3B siRNA 2 in MDA-MB-231 and MDA-MB-436 cells. Knockdown of DNMT3B by siRNA reduced proliferation, migration, and invasion of MDA-MB-231 and MDA-MB-436 cells, respectively (Fig. 6e–h, Additional file 1: Figure S8). These results suggest that DNMT3B played an important role in the functions of breast cancer cells.

## Discussion

Breast cancer is a heterogeneous disease, both biologically and clinically. More and more evidence supports the notion that microRNAs (miRNAs) play a critical role as oncogenes or as tumor suppressor genes in cancers. miR-29c is significantly downregulated in many cancers including breast cancer, suggesting that miR-29c acts as a suppressor miRNA in cancers. However, the functional role and underlying mechanism of miR-29c action in breast cancer have not been elucidated. In this study, we investigated the function of miR-29c and explored its underlying mechanism in breast cancers. We firstly reported that miR-29c plays a significant role in suppressing the progression of breast cancers by targeting the TIMP3/STAT1/FOXO1 pathway. Our results also showed there was a lower miR-29c expression in breast cancers than that in normal tissues and that overexpression of miR-29c inhibited cell proliferation, migration and invasion of cells, colony formation, and growth in 3D Matrigel. In addition, we identified that miR-29c directly targets DNMT3B and inhibited expression of DNMT3B which was associated with methylation of TIMP3.

It was reported that miR-29c-5p expression was upregulated in breast cancers with the luminal subtypes [34]. Elizabeth Poli et al. reported that MicroRNA-29c (miR-29c) has been shown to be significantly downregulated in basal-like breast tumors and to be involved in cell invasion and sensitivity to chemotherapy [16]. Study showed that there was significantly reduced expression of miR-29c in basal-like breast cancers compared to other breast cancer molecular subtypes and that miR-29c was associated with DNMT3B and methylation of genes [17]. Study showed that miR-29c efficiently downregulated B7-H3 expression and the expression of miR-29c correlated with survival rate of breast cancer patients, suggesting a tumor suppressive role of miR-29c [35]. Our results showed that there was a different expression level of miR-29c in breast cancer cells with different subtypes. MCF-7 is a breast cancer cell line with ER positive. MDA-MB-231 is a breast cancer cell line with ER negative, PR negative, and HER2 negative). Our results further confirmed that the expression of miR-29c was associated with the subtype of breast cancers, grades of cancer, methylation of genes in breast cancers, and survival rate of patients with breast cancers. miR-29c was found to exert a suppressive role in the development of breast cancers. These results can be very useful to identify miR-29c as a biomarker of diagnosis and therapy in breast cancers.

DNA methyltransferases, including DNMT1, DNMT3A, and DNMT3B, are enzymes that catalyze DNA methylation. DNMT3A and DNMT3B are mainly involved in de novo DNA methylation, whereas DNMT1 is required for the maintenance of pre-existing methylation [36]. In cancer tissues, DNMT3B is expressed more frequently compared to DNMT1 and DNMT3A [37]. In breast cancer, DNMT3B is also frequently overexpressed [24, 25]. We did in silico analysis using the Oncomine database (www.oncomine.org) and found that the expression level of DNMT3B was higher in invasive ductal breast carcinomas than in normal tissues, confirming previous results. miRNAs, acting as post-transcriptional regulators, can directly degrade target mRNAs and/or repress their translation in a sequence-specific manner [38]. Our previous study showed that miR-29c was negatively correlated with the expression of DNMT3B in melanoma [18]. Here, we demonstrated that miR-29c targeted the 3′UTR of DNMT3B and inhibited its expression, and that both miR-29c expression and DNMT3B expression were associated with the survival rate of patients with breast cancer.

Methylation of gene promoters increases with the progression of cancers [39, 40]. DNMT3B is overexpressed and is involved in the methylation of genes in cancers [18]. One of the major mechanisms of the carcinogenesis process is thought to be the inactivation of tumor suppressor genes by the methylation of their promoter regions. Abnormal expression of DNMT3B is related to the hypermethylation of the tumor suppressor genes. TIMP3 is an inhibitor of extracellular matrix metalloproteinase that can suppress angiogenesis [41, 42], tumor growth [43, 44], and invasion and migration [43–45]. It has been reported that in many common tumors, CpG islands of TIMP3 undergo methylation frequently [46, 47] and that in the primary tumors, the methylation of the TIMP3 promoter can lead to the loss of its protein expression [48]. Moreover, treating methylated human gastric cancer cell lines with 5-aza can even rescue the defective expression of TIMP3 [49]. These studies suggest that epigenetic changes in tumors may play an important role in regulating TIMP3 expression [50]. miR-29c regulated DNMT3B which was associated with methylation of genes in breast cancers [17]. There is a lower level of miR-29c expression in hypermethylator breast cancer cell lines than non-hypermethylator breast cancer cell lines. The expression of miR-29c correlated inversely with methylation-sensitive gene expression and directly with the methylation status of these genes [31]. In this study, methylation of the TIMP3 promoter and changes in expression of TIMP3 and DNMT3B were all affected by miR-29c regulation. In addition, the direct binding of miR-29c to the wild-type 3′UTR of DNTM3B indicates that miR-29c is the direct post-transcriptional regulator of DNMT3B, which is elevated in breast cancer and methylates the promoter of the TIMP3 gene. Taken together, our results support the concept that the expression of TIMP3 is ultimately regulated by miR-29c.

Signal transducer and activator of transcription 1 (STAT1) is a member of the STAT protein family, which plays important roles in cancer inflammation. STAT1 was associated with cancers, especially in breast cancers [51]. In tumor microenvironment, tumor-induced stromal STAT1 increased the progression of breast cancer via deregulating tissue homeostasis [52]. STAT1 can promote the growth of breast cancer by inhibiting immunity [53]. Inhibition of STAT1 signaling reduced the primary growth and progression of breast cancer cells [54]. When activated via phosphorylation at the Tyr701 site, STAT1 binds specific regulatory elementary and regulates the transcription of its target genes [55]. Forkhead box protein O1 (FOXO1) belongs to the forkhead family of transcription factors which are characterized by a distinct forkhead domain and play many important roles in cancers [56]. FOXO1 was considered as tumor suppressor gene. The RNA-binding protein Quaking (QKI) resulted in low levels of FOXO1 expression in breast cancer cells [57]. Astrocyte-elevated gene-1 (AEG-1) inhibited the expression of FOXO1 and promoted the progression of breast cancer [58]. Acylglycerol kinase was reported to promote the growth of cells and tumorigenicity in breast cancer by inhibiting the expression of FOXO1 [59].TIMP3 is a versatile extracellular regulator in cancers [60]. The loss of TIMP3 can lead to diabetic kidney disease in both human and mouse via the interplay of FOXO1 and STAT1 [61]. In addition, miR-29c is involved in diabetic nephropathy by targeting tristetraprolin [62]. However, it is unclear that TIMP3 inhibits the progress of breast cancer via the FOXO1/STAT1 pathway. We show that the expression of TIMP3 and FOXO1 is decreased whereas the expression of STAT1 is increased in miR-29c knockdown MCF-7 cells. STAT1 has been reported to exhibit a negative regulatory effect on FOXO1 transcription in pancreatic β cells [61] and bladder cancer [63]. Fludarabine is a specific inhibitor of STAT1 [64]. Fludarabine inhibited the decrease of FOXO1 expression caused by knockdown of miR-29c and abrogated the increment of cell migration and invasion caused by knockdown of miR-29c. We firstly illustrated that miR-29c exerted its regulatory function in breast cancers by activating the TIMP3/STAT1/FOXO1 pathway. This is firstly reported that miR-29c inhibited the proliferation, migration, invasion colony, and 3D growth of breast cancer cells by targeting TIMP3/STAT1/FOXO1 pathway.

## Conclusion

**Fig. 7:**
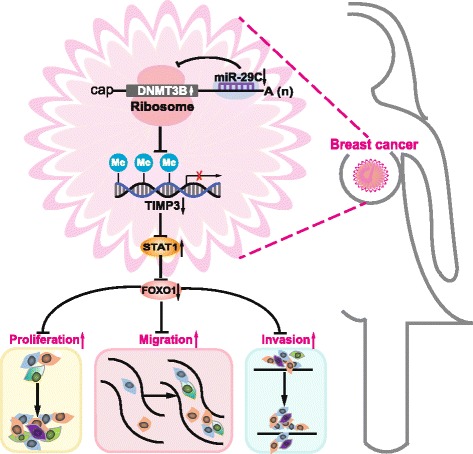
Proposed mechanistic scheme: miR-29c suppresses breast cancer by the TIMP3/STAT1/FOXO1 pathway. The expression of miR-29c was decreased in breast cancer, which leading to the elevated expression of DNMT3B that is critical for promoter methylation and decreases the expression of TIMP3. As a result, the expression of STAT1 was increased, and it inhibits the expression of FOXO1, leading to proliferation, migration, and invasion of breast cancer cells

In conclusion, we found that the expression of miR-29c is correlated with the progress and prognosis of breast cancers. DNMT3B is a direct target of miR-29c. DNMT3B expression increases as miR-29c expression decreases in cells. Upregulated DNMT3B is critical for promoter methylation and decreased expression of TIMP3, which could promote progression of breast cancer via the TIMP3/STAT1/FOXO1 pathway (Fig. 7). These results suggest that miR-29c could be used as a potential biomarker of diagnosis and therapy in breast cancers.

## Additional file

Additional file 1: **Table S1.** Sequence of DNMT3B siRNA. **Table S2.** Primers of miR-29c and DNMT3B. **Table S3.** Primers of TIMP3 for methylation specific PCR and unmethylation PCR. **Figure S1.** Quantification of protein expression level of DNMT3B in human breast cancer tissues and the paired adjacent non-tumor tissues. **Figure S2.** Migration and invasion of cells. **Figure S3.** miR-29c inhibited proliferation, migration and invasion, colony formation and growth in 3D Matrigel of MDA-MB-436 cells. **Figure S4.** DNMT3B promoted migration, invasion, colony formation and growth in 3D Matrigel of MDA-MB-231 miR-29c cells. **Figure S5.** Colony formation of cells. **Figure S6.** miR-29c reduced luciferase activity of wild type 3’ UTR of DNMT3B-luciferase reporter, and not the mutant type 3’ UTR of DNMT3B reporter in MCF-7 cells. **Figure S7.** Expression of DNMT3B, TIMP3, STAT1 and FOXO1. **Figure S8.** Migration and invasion of cells. (DOCX 3215 kb)

## Abbreviations

DNMT: DNA methyltransferases
FBS: Fetal bovine serum
FOXO1: Forkhead box protein O1
IHC: Immunohistochemistry
MTT: 3-(4,5-Dimethylthiazol-2-yl)-2, 5-diphenyltetrazolium bromide
qRT-PCR: Quantitative real-time polymerase chain reaction
STAT1: Signal transducer and activator of transcription 1
STR: Short tandem repeat
TIMP3: Tissue inhibitor of metalloproteinases 3
UTR: Untranslated region
