# Correction: miR-29c plays a suppressive role in breast cancer by targeting the TIMP3/STAT1/FOXO1 pathway

**DOI:** 10.1186/s13148-022-01317-4

**Published:** 2022-07-29

**Authors:** Wan Li, Jie Yi, Xiangjin Zheng, Shiwei Liu, Weiqi Fu, Liwen Ren, Li Li, Dave S. B. Hoon, Jinhua Wang, Guanhua Du

**Affiliations:** 1 The State Key Laboratory of Bioactive Substance and Function of Natural Medicines, Beijing, China; 2 Key Laboratory of Drug Target Research and Drug Screen, Institute of Materia Medica, Chinese Academy of Medical Science and Peking Union Medical College, Beijing, 100050 China; 3 Department of Clinical Laboratory, Peking Union Medical College Hospital, Beijing, 100730 China; 4 Department of Endocrinology, Shanxi DAYI Hospital, Shanxi Medical University, Taiyuan, 030002 Shanxi China; 5 Department of Translational Molecular Medicine, John Wayne Cancer Institute (JWCI) at Providence Saint John’s Health Center, Santa Monica, CA 90404 USA

## Correction: Clinical Epigenetics (2018) 10:64 https://doi.org/10.1186/s13148-018-0495-y

Following publication of the original article [1], the authors noticed the errors in Fig. 4b and Fig. S4A in the supplementary material. The revised Fig. 4 has been presented with this erratum and the revised supplementary material with the inclusion of new Fig. S4A has been uploaded.

**Fig. 4:**
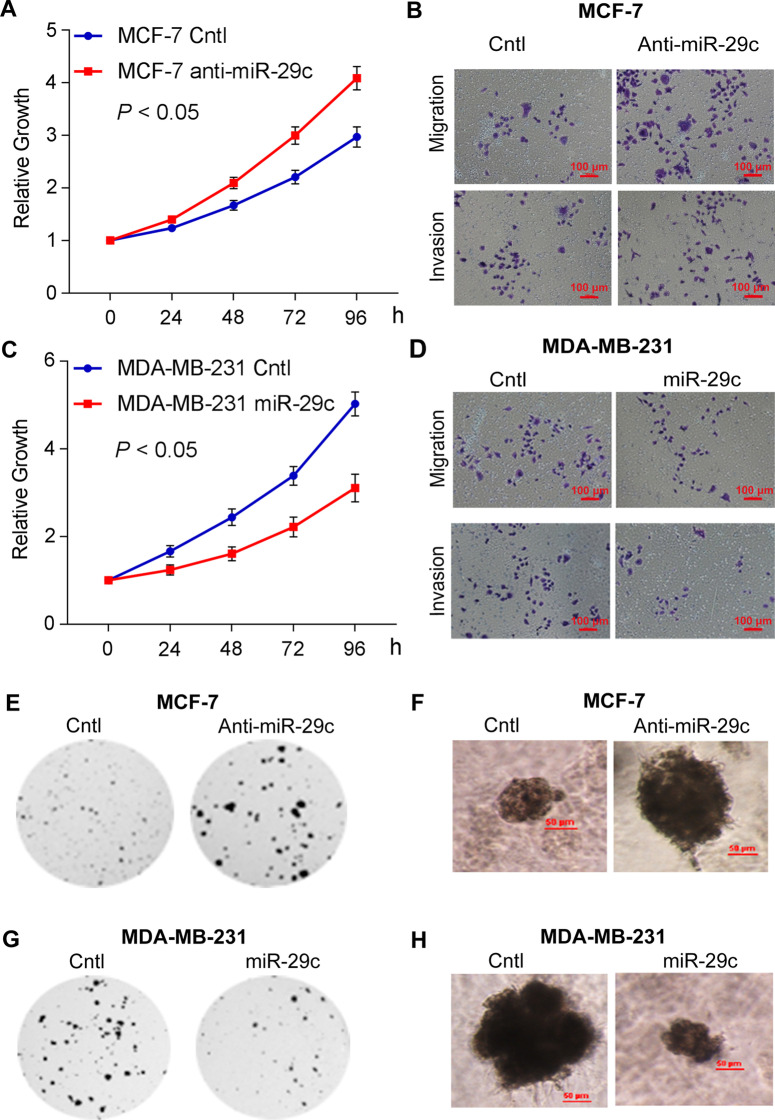
miR-29c inhibited the proliferation, migration and invasion, colony formation, and growth in 3D Matrigel of breast cancer cells. **a** Proliferation ofMCF-7 anti-miR-29c is higher than that of MCF-7 Cntl by CCK8 proliferation assay. **b** Migration and invasion of MCF-7 anti-miR-29cis higher than that ofMCF-7 Cntl. **c** Proliferation of MDA-MB-231 miR-29c mimic is lower than that MDA-MB-231 Cntl by CCK8 proliferation assay. **d** Migration and invasion assays of MDA-MB-231 miR-29c mimic are lower than that MDA-MB-231 Cntl. **e** Colony formations of MCF-7 anti-miR-29c are more than that of MCF-7Cntl in Soft agar assays. **f** Growth of MCF-7 anti-miR-29c is more than that of MCF-7 Cntl in 3D Matrigel culture. **g** Colony formations of MDA-MB-231miR-29c mimic are less than that of MDA-MB-231 Cntl in soft agar assays. **h** Growth of MDA-MB-231 miR-29c mimic is less than that of MDA-MB-231Cntl in 3D Matrigel culture. Data are presented as mean ± SD from three independent experiments, and every experiment was repeated three times, *P < 0.05

## Supplementary Information

**Additional file 1: Table S1.** Sequence of DNMT3B siRNA. **Table S2.** Primers of miR-29c and DNMT3B. **Table S3.** Primers of TIMP3 for methylation specific PCR and unmethylation PCR. **Figure S1.** Quantification of protein expression level of DNMT3B in human breastcancer tissues and the paired adjacent non-tumor tissues. **Figure S2.** Migration and invasion of cells. **Figure S3.** miR-29c inhibited proliferation, migration and invasion, colony formation and growth in 3D Matrigel of MDA-MB-436 cells. **Figure S4.** DNMT3B promoted migration, invasion, colony formation and growth in 3D Matrigel of MDA-MB-231 miR-29c cells. **Figure S5.** Colony formation of cells. **Figure S6.** miR-29c reduced luciferase activity of wild type 3’UTR of DNMT3B-luciferase reporter, and not the mutant type 3’UTR of DNMT3B reporter in MCF-7 cells. **Figure S7.** Expression of DNMT3B, TIMP3, STAT1 and FOXO1. **Figure S8.** Migration and invasion of cells.
