# Supplementary material for: Correction: miR-29c plays a suppressive role in breast cancer by targeting the TIMP3/STAT1/FOXO1 pathway
Source: Clin Epigenetics. 2022 Jul 29;14:97. doi: 10.1186/s13148-022-01317-4 (PMC9338476; doi:10.1186/s13148-022-01317-4)
Supplement: Supplementary file 1 — Additional file 1: Table S1. Sequence of DNMT3B siRNA. Table S2. Primers of miR-29c and DNMT3B. Table S3. Primers of TIMP3 for methylation specific PCR and unmethylation PCR. Figure S1. Quantification of protein expression level of DNMT3B in human breastcancer tissues and the paired adjacent non-tumor tissues. Figure S2. Migration and invasion of cells. Figure S3. miR-29c inhibited proliferation, migration and invasion, colony formation and growth in 3D Matrigel of MDA-MB-436 cells. Figure S4. DNMT3B promoted migration, invasion, colony formation and growth in 3D Matrigel of MDA-MB-231 miR-29c cells. Figure S5. Colony formation of cells. Figure S6. miR-29c reduced luciferase activity of wild type 3’UTR of DNMT3B-luciferase reporter, and not the mutant type 3’UTR of DNMT3B reporter in MCF-7 cells. Figure S7. Expression of DNMT3B, TIMP3, STAT1 and FOXO1. Figure S8. Migration and invasion of cells. [file 13148_2022_1317_MOESM1_ESM.docx]

**Additional file**

**miR-29c plays suppress roles in breast cancer by activating**

**TIMP3/STAT1/FOXO1 pathway**

**Wan Li**^1,2,*^**, Jie Yi**^3*^**，Xiangjin Zheng**^1,2^**, Shiwei Liu**^4^,**Weiqi Fu**^1,2^**, Li Li** ^2^**, Dave Hoon**^5^**, Jinhua Wang**^1,2,£^**, Guanhua Du**^1,2,£^

^1^The State Key Laboratory of Bioactive Substance and Function of Natural Medicines;

^2^Key Laboratory of Drug Target Research and Drug Screen, Institute of Materia Medica, Chinese Academy of Medical Science and Peking Union Medical College. Beijing, China 100050.

^3^Department of Clinical Laboratory, Peking Union Medical College Hospital, Beijing, China 100730.

^4^Department of Endocrinology, Shanxi DAYI Hospital, Shanxi Medical University, Taiyuan, Shanxi, China, 030002.

^5^ Department of Molecular Oncology, John Wayne Cancer Institute (JWCI) at Providence Saint John’s Health Center, Santa Monica, CA 90404.

^*^ These authors contributed equally to this work.

^£^Both authors should be considered as corresponding authors:

Jinhua Wang, Email: wjh@imm.ac.cn, Fax:86-10-63165184

Guanhua Du, Email: dugh@imm.ac.cn, Fax:86-10-63165184

**Table S1: Sequence of DNMT3B siRNA**

| DNMT3B siRNA | Sequence |
| --- | --- |
| siRNA 1 | Sense strand, 5’-AGAUGACGGAUGCCUAGAGUUCCU GUCUC A-3’ |
|  | Anti-sense strand, 5’-GCUCUAGGCAUCCGUCAUCUUU CCUGUCUC -3’ |
| siRNA 2 | Sense strand, 5’- UCUGUCCGUUCACAUGUGUUU-3’ |
|  | Anti-sense strand, 5’-GUACACAUGUGAACGGACAGAUU-3’ |

**Table S2: Primers of miR-29c and DNMT3B**

| Gene name | Primer sequence |
| --- | --- |
| miR-29c | Forward: 5’-CTGACCTTAGCACCATTTGAAATC-3’ |
|  | Reverse: 5’-TATCGTTGTACTCCACTCCTTGAC-3’ |
| DNMT3B | Forward: 5’-AGGGAAGACTCGATCCTCGTC-3’ |
|  | Reverse: 5’-GTGTGTAGCTTAGCAGACTGG-3’ |

**Table S3: Primers of TIMP3 for methylation specific PCR and unmethylation PCR**

| Primer type | Primer sequence |
| --- | --- |
| M primer | Forward: 5’-TCGAGAGATAGAAATATTTTTACGA-3’ |
|  | Reverse: 5’-TAACATTAAAACAACAAAAACCGAA-3’ |
| U primer | Forward: 5’-TTGAGAGATAGAAATATTTTTATGA-3’ |
|  | Reverse: 5’-TAACATTAAAACAACAAAAACCAAA-3’ |

**
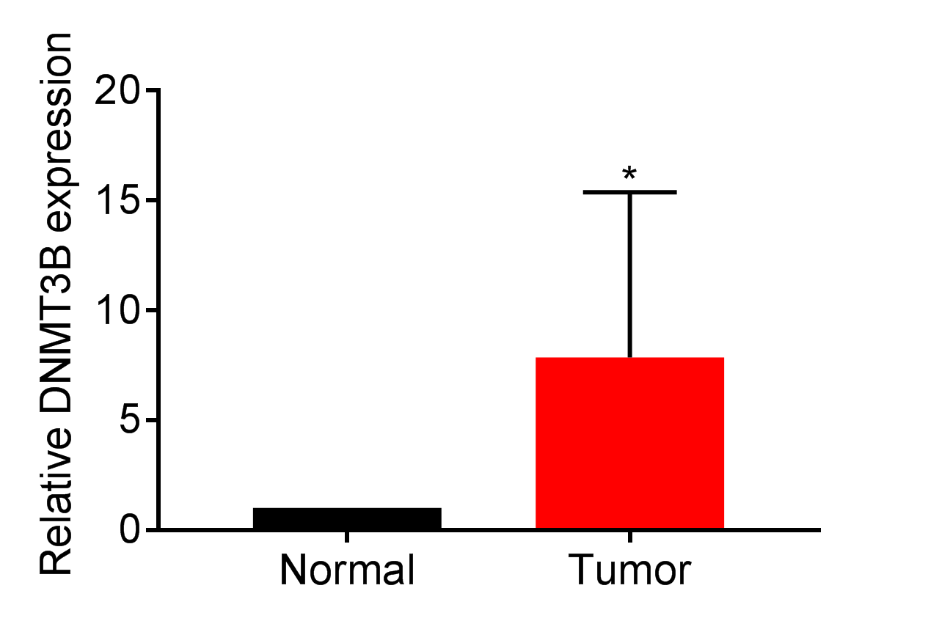
**

**Figure S1. Quantification of protein expression level of DNMT3B in human breast cancer tissues and the paired adjacent non-tumor tissues.** Data were presented as mean ± SD, n = 8, **P* < 0.05.

**
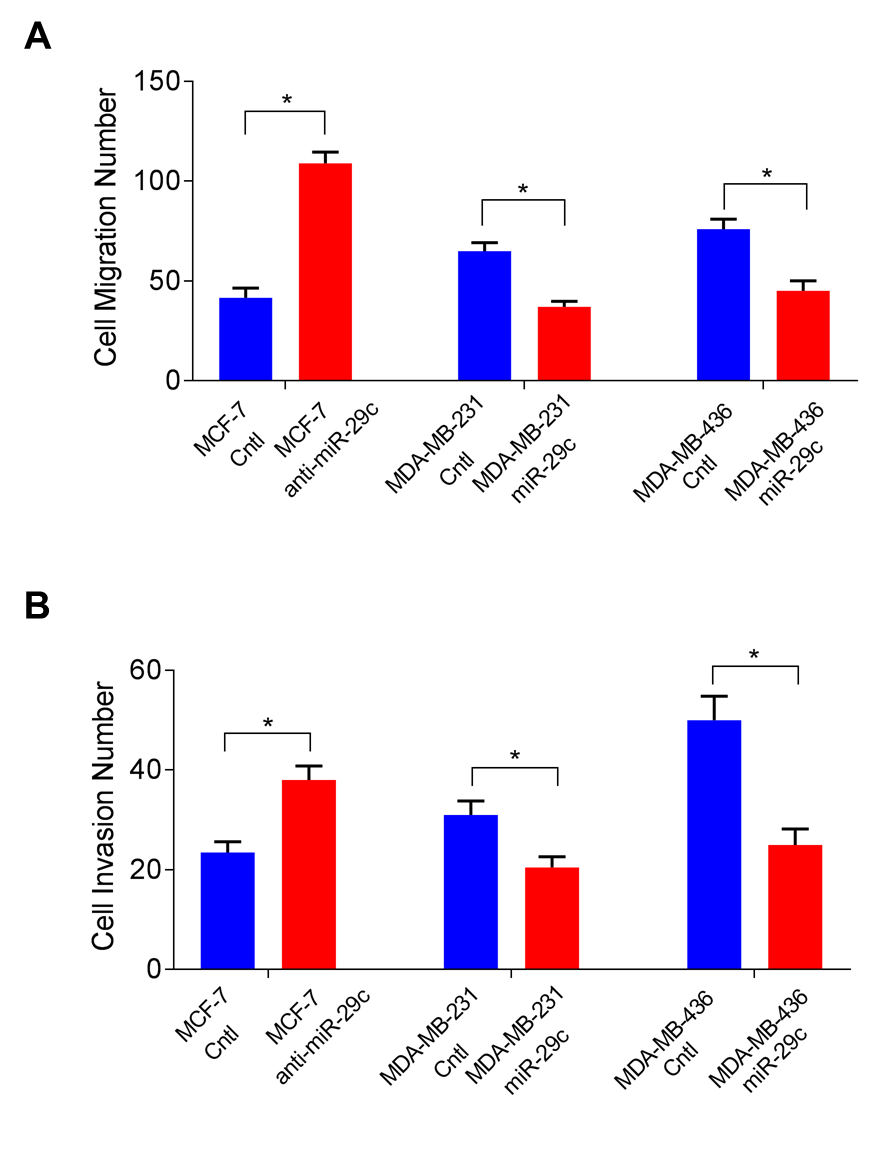
**

**Figure S2. Migration and invasion of cells.**

**A:** Quantification of migration number of MCF-7 Cntl and MCF-7 transfected with anti-miR-29c, MDA-MB-231 Cntl and MDA-MB-231 transfected with mimic-miR-29c, MDA-MB-436 Cntl and MDA-MB-436 transfected with mimic-miR-29c. **B.** Quantification of invasion number of MCF-7 Cntl and MCF-7 transfected with anti-miR-29c, MDA-MB-231 Cntl and MDA-MB-231 transfected with mimic-miR-29c, MDA-MB-436 Cntl and MDA-MB-436 transfected with mimic-miR-29c. Data are presented as mean ± SD. from three independent experiments, and every experiment was repeated three times, **P* < 0.05.


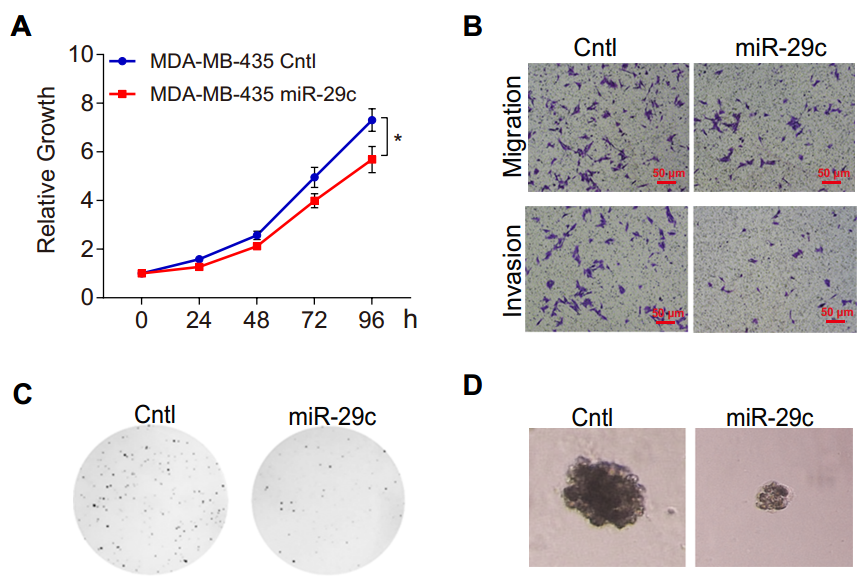

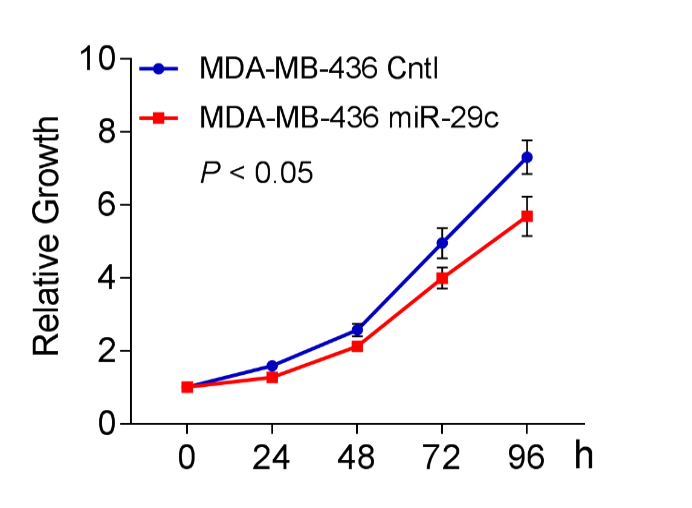


**Figure S3. miR-29c inhibited proliferation, migration and invasion, colony formation and growth in 3D Matrigel of MDA-MB-436 cells.** **A.** CCK8 proliferation assays of MDA-MB-436 cells after overexpression of miR-29c. **B.** Migration and invasion assays of MDA-MB-436 cells after overexpression of miR-29c. **C.** Soft agar colony formation assays in MDA-MB-436 cells after overexpression of miR-29c. **D.** 3D Matrigel culture of MDA-MB-436 cells after overexpression of miR-29c. Data are presented as mean ± SD. from three independent experiments, and every experiment was repeated three times.

**
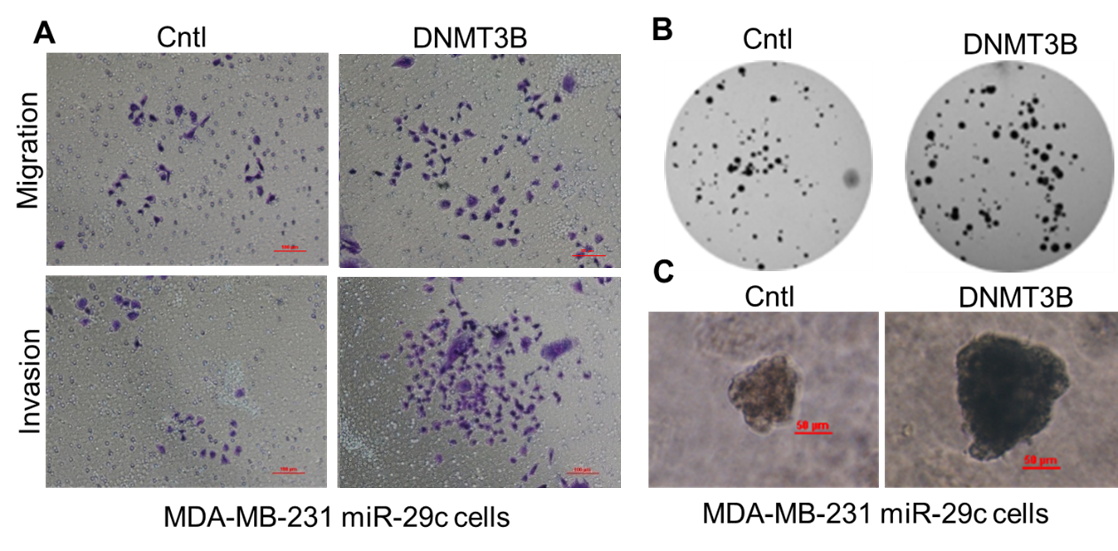
**

**Figure S4. DNMT3B promoted migration, invasion, colony formation and growth in 3D Matrigel of** **MDA-MB-231 miR-29c cells. A.** Migration and invasion assays of MDA-MB-231 miR-29c cells after the overexpression of DNMT3B. **B.** Soft agar colony formation assays in MDA-MB-231 miR-29c cells after the overexpression of DNMT3B. **C.** 3D Matrigel culture of MDA-MB-231 miR-29c cells after the overexpression of DNMT3B. Data are presented as mean ± SD. from three independent experiments, and every experiment was repeated three times.


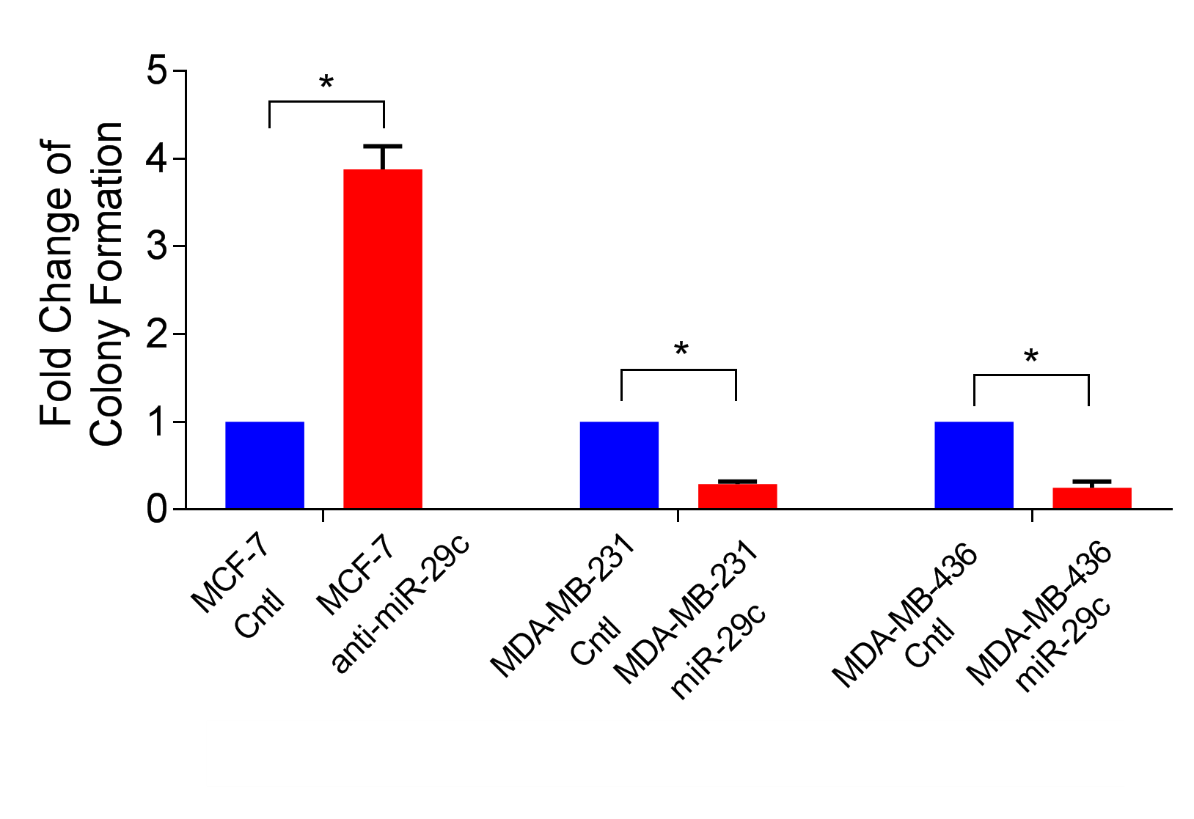


**Figure S5. Colony formation of cells**

Quantification of colony formation of MCF-7 Cntl and MCF-7 transfected with anti-miR-29c, MDA-MB-231 Cntl and MDA-MB-231 transfected with mimic-miR-29c, MDA-MB-436 Cntl and MDA-MB-436 transfected with mimic-miR-29c. Data are presented as mean ± SD. from three independent experiments, and every experiment was repeated three times, *P < 0.05.

**
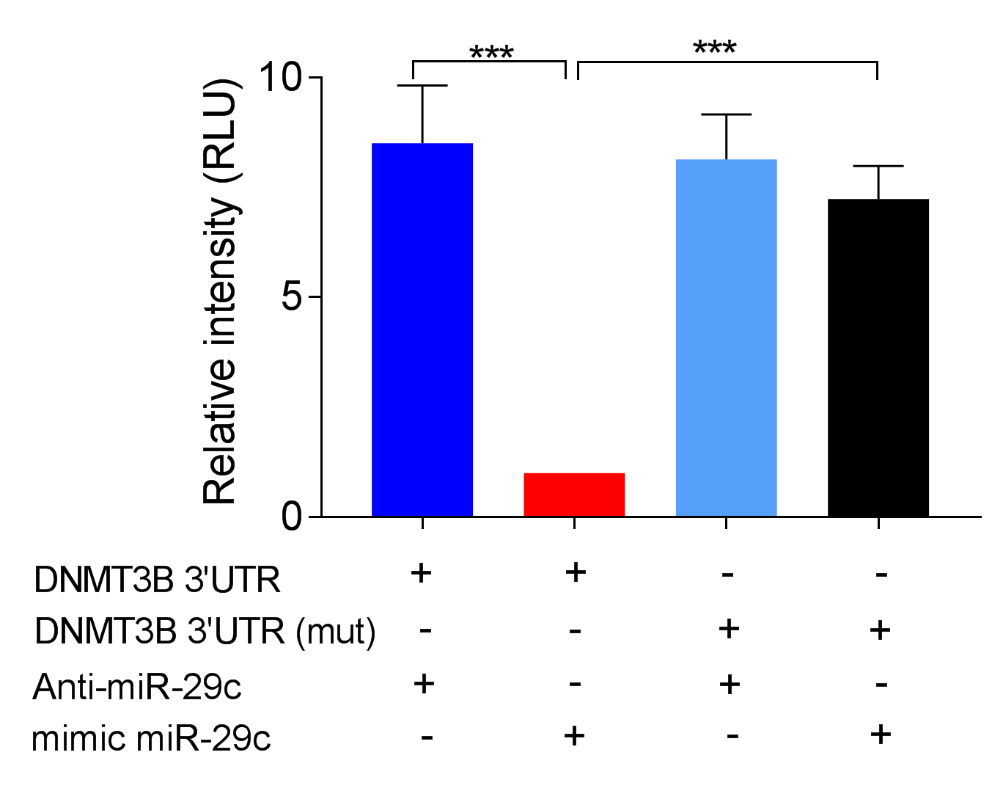
**

**Figure S6. miR-29c reduced luciferase activity of wild type 3’ UTR of DNMT3B-luciferase reporter, and not the mutant type 3’ UTR of DNMT3B reporter in MCF-7 cells**. Data are presented as mean ± SD. from three independent experiments, ****P* < 0.001.

**
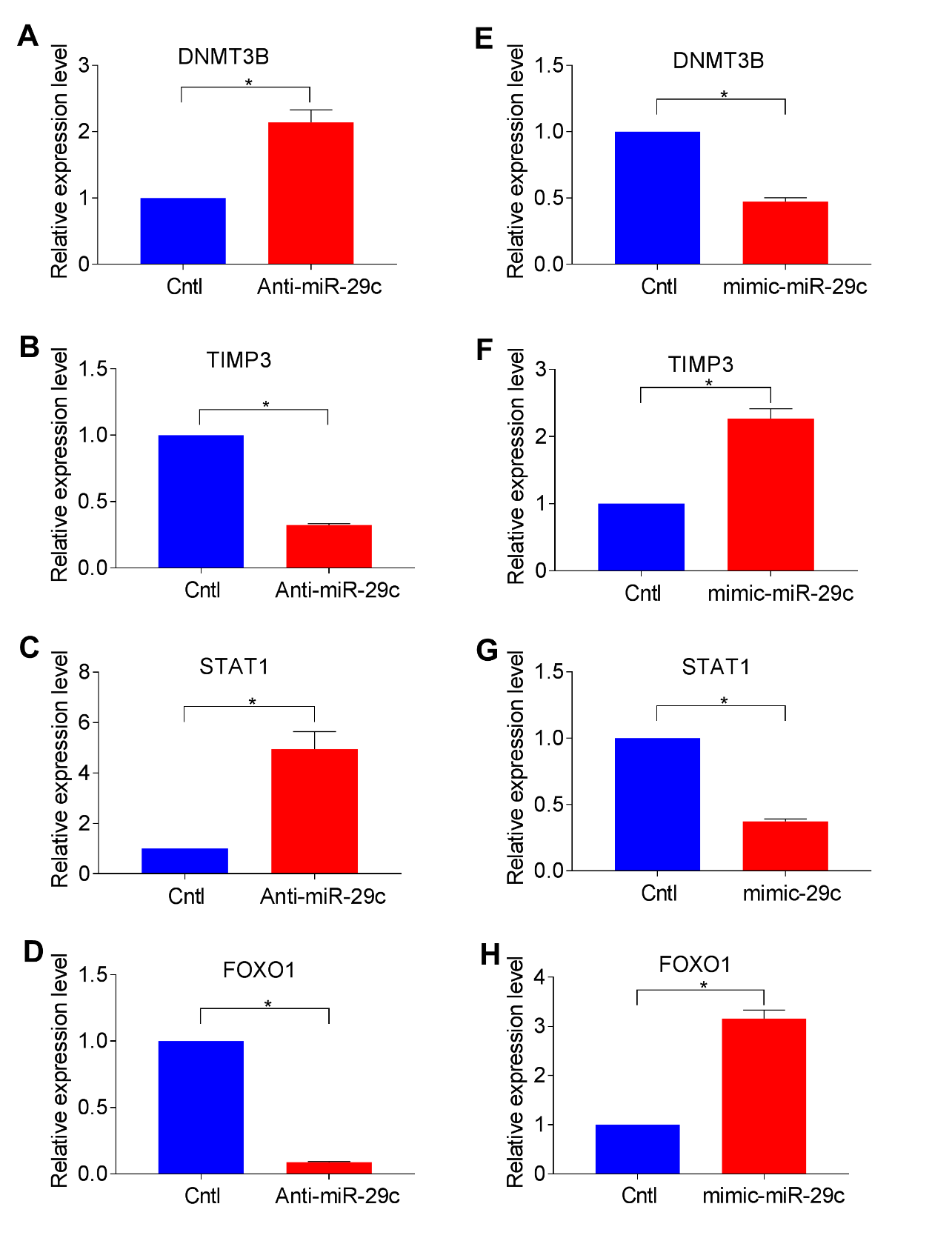
**

**Figure S7. Expression of DNMT3B, TIMP3, STAT1 and FOXO1.**

Quantification of protein expression of DNMT3B, TIMP3,STAT1 and FOXO1 in MCF-7 Cntl and MCF-7 transfected with anti-miR-29c, MDA-MB-231 Cntl and MDA-MB-231 transfected with mimic-miR-29c. Data are presented as mean ± SD. from three independent experiments, and every experiment was repeated three times, **P* < 0.05.


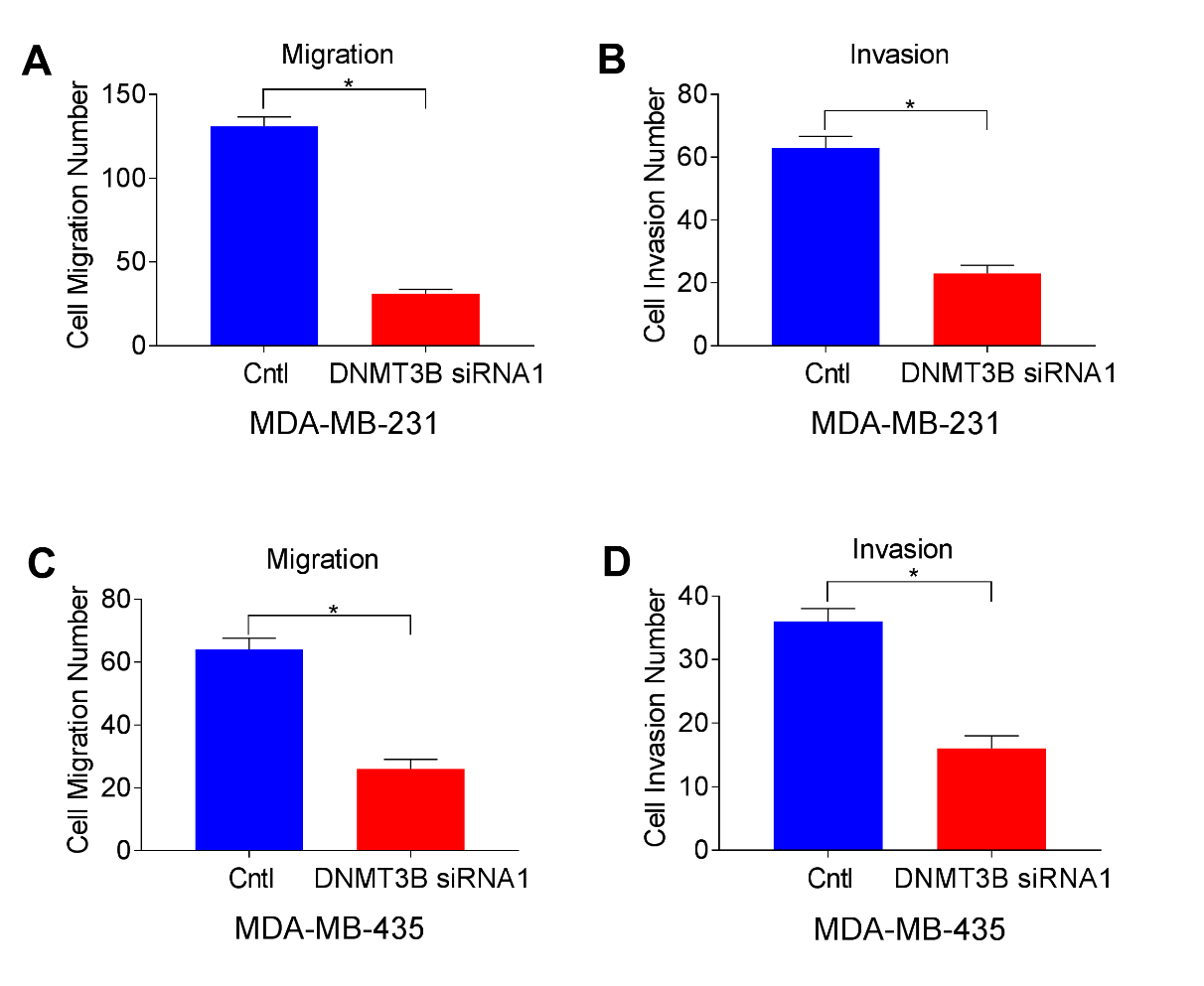


**Figure S8. Migration and invasion of cells. A.** Quantification of migration number of MDA-MB-231 Cntl and MDA-MB-231 transfected with DNMT3B siRNA 1. **B**. Quantification of invasion number of MDA-MB-231 Cntl and MDA-MB-231 transfected with DNMT3B siRNA 1. **C**. Quantification of migration number of MDA-MB-231 Cntl and MDA-MB-436transfected with DNMT3B siRNA 1. **D.** Quantification of invasion number of MDA-MB-436 Cntl and MDA-MB-436 transfected with DNMT3B siRNA1. Data are presented as mean ± SD. from three independent experiments, and every experiment was repeated three times, **P* < 0.05.
